# Prevalence and Metabolic Characterization of Polycystic Ovary Syndrome in a Cohort of Patients Diagnosed with Spina Bifida: Study Protocol

**DOI:** 10.3390/children12070851

**Published:** 2025-06-27

**Authors:** Giorgio Sodero, Clelia Cipolla, Federica Arzilli, Claudia Rendeli

**Affiliations:** 1 Department of Life Sciences and Public Health, Fondazione Policlinico Universitario A. Gemelli IRCCS, 00168 Rome, Italy; 2 Pediatric Department, Perrino Hospital, 72100 Brindisi, Italy; 3 Pediatric Endocrinology Unit, Perrino Hospital, 72100 Brindisi, Italy; 4 Spina Bifida Unit, Fondazione Policlinico Universitario A. Gemelli IRCCS, 00168 Rome, Italy; 5 Università Cattolica Sacro Cuore, 00168 Rome, Italy

**Keywords:** spina bifida, polycystic ovary syndrome, pediatric endocrinology, pediatric urology

## Abstract

**Background:** Myelomeningocele, commonly known as spina bifida, is a congenital malformation of the spinal cord. Polycystic ovary syndrome (PCOS) is a complex endocrine-metabolic disorder affecting 9–21% of women of reproductive age and is characterized by hyperandrogenism and ovulatory dysfunction. Hyperhomocysteinemia, insulin resistance, metabolic syndrome, and alterations in inositol metabolism play a crucial pathophysiological role in both PCOS and spina bifida; however, the potential link between these two significant conditions has not been explored. **Objectives:** The primary objective of our study is to assess the prevalence of PCOS among female pediatric patients with a prior diagnosis of spina bifida. Additionally, we will evaluate differences in auxological and metabolic parameters between patients diagnosed with PCOS and those without the diagnosis. The secondary objectives of our study include the following: characterizing the metabolic profiles of patients with PCOS and differentiating between various phenotypic forms of PCOS. **Methods:** Prospective, cross-sectional, observational, and monocentric study. The study will have an overall duration of 24 months, with the potential for extension until the last patient is enrolled. The recruitment period is set at 12 months.

## 1. Introduction

### 1.1. Background

Myelomeningocele, commonly known as spina bifida, is a congenital malformation in which the spinal cord does not develop properly due to the incomplete closure of the neural tube around the 28th day of gestation [1]. It is the most common congenital defect affecting the central nervous system and leads to permanent disability. Spina bifida occurs in 1–10 out of 1000 live births worldwide [2]. Although it is considered a rare condition, with a significantly reduced incidence due to prevention strategies and the possibility of fetal surgical correction [1,2], there are currently many children and young adults affected by this condition and other forms of spinal dysraphism who require follow-up and regular clinical evaluations.

The management of myelomeningocele requires comprehensive lifelong neurological, urological, musculoskeletal, and dermatological care; these patients have mobility issues and are subjected to multiple pharmacological treatments for associated urinary dysfunctions and the risk of complicated urinary tract infections [1,2,3]. Approximately 90–95% of affected infants are born to parents with no family history of spina bifida. Among the non-genetic factors associated with spina bifida are environmental exposures, reduced dietary folate intake, hyperthermia, maternal anticonvulsant therapy (valproic acid and carbamazepine), pregestational maternal diabetes mellitus, and obesity [4]. Another critical micronutrient for neural tube closure, after folic acid, is inositol, which has shown efficacy in preventing neural tube defects (NTDs) in folic acid-resistant mice [5].

Inositol is a carbocyclic polyol sugar that serves as a precursor for several second messengers critical for signal transduction in the brain, kidneys, reproductive organs, and other tissues in response to neurotransmitters, hormones, and growth factors [6,7,8]. Altered inositol concentrations have been observed in various diseases, including neural tube defects, metabolic syndrome, polycystic ovary syndrome (PCOS), and diabetes [6].

Inositol also plays a role in insulin-dependent processes as insulin second messengers [9]. MI is converted into an insulin second messenger inositol phosphoglycan (MI-IPG) and is involved in cellular glucose uptake, while DCI is converted into an insulin second messenger IPG (DCI-IPG) and contributes to glycogen synthesis [10,11,12].

Polycystic ovary syndrome (PCOS) is a complex endocrine-metabolic disorder affecting 9–21% of women of reproductive age and is characterized by hyperandrogenism and ovulatory dysfunction. According to the consensus of the European Society of Human Reproduction and Embryology (ESHRE)/American Society of Reproductive Medicine (ASRM) [13], diagnosis is based on the presence of at least two of the following Rotterdam criteria:

(a) Oligo-ovulation and/or anovulation;
(b) Clinical and/or biochemical signs of hyperandrogenism;
(c) Ultrasound evidence of polycystic ovaries, excluding other etiologies.

It is estimated that a significant proportion of PCOS patients (50–60%) exhibit insulin resistance, and the prevalence of metabolic syndrome in women with PCOS is approximately 24%.

Some studies on adults with spinal cord injury have shown that cardiovascular disease is the leading cause of death in these patients, with an increased risk of morbidity and mortality compared to healthy individuals. Evidence also suggests a higher prevalence of undiagnosed symptomatic coronary disease in patients with spinal cord injury [14,15,16,17,18,19,20,21,22,23,24,25,26,27,28,29,30,31,32]. Key indicators of increased cardiovascular risk include low high-density lipoprotein (HDL) cholesterol levels, likely due to an inactive lifestyle and other genetic, behavioral (smoking, alcohol consumption, and activity level), age, lesion level, time since injury, and anthropometric factors (body mass or adipose tissue) [33].

### 1.2. Homocysteine (Hcy) and Cardiovascular Risk

Homocysteine (Hcy) is also considered an important cardiovascular risk factor. Elevated Hcy levels in peripheral blood can lead to vascular diseases, coronary dysfunction, atherosclerotic changes, and embolic diseases [26]. The Framingham Offspring Study demonstrates that hyperhomocysteinemia is associated with hyperinsulinemia and may partially account for the increased cardiovascular risk associated with insulin resistance [29].

Recent studies have assessed Hcy levels in the PCOS population, showing a statistically significant increase in Hcy levels among women with PCOS who exhibit insulin resistance, androgen excess, elevated cardiovascular risk markers, recurrent pregnancy loss, and those undergoing metformin treatment. An increased prevalence of cardiovascular disease and higher cardiovascular morbidity has also been reported in women with PCOS [27,28].

### 1.3. PCOS Pathophysiology and Correlation with Spina Bifida

The pathophysiology of PCOS is multifaceted, involving several factors such as the dysfunction of the hypothalamic–pituitary–ovarian axis, which is caused by defects in steroidogenesis, insulin resistance, fat deposition, especially in the abdominal region, and hyperandrogenism [34]. The pathogenesis of menstrual disorders related to PCOS is still not fully understood, as animal studies have shown that prolonged dehydroepiandrosterone therapy does not cause PCOS [35], despite the presence of known hyperandrogenism in PCOS [1].

The potential correlation between spina bifida and polycystic ovary syndrome has not yet been explored in any prospective study; currently, there are no data on the incidence and prevalence of this condition in women with spinal dysraphisms.

A potential correlation between spinal dysraphisms and PCOS could exist, given the presence of common risk factors and similar alterations in homocysteine and inositol metabolism; however, these are merely assumptions, as there is currently no scientific evidence on the matter.

Based on these observations, we propose this prospective cross-sectional single-center study to determine the prevalence as well as the phenotypic and metabolic characteristics of PCOS in female patients with spina bifida.

## 2. Materials and Methods

### 2.1. Objectives

The primary objective of our study is to assess the prevalence of PCOS among female patients with a prior diagnosis of spina bifida. Additionally, we will evaluate differences in auxological and metabolic parameters between patients diagnosed with PCOS and those without the diagnosis.

The secondary objectives of our study include the following:

- Characterizing the metabolic profile of patients with PCOS.
- Differentiating between various phenotypic forms of PCOS.

### 2.2. Study Design

This is a prospective, cross-sectional, observational study. The study will have an overall duration of 24 months, with the potential for extension until the last patient is enrolled. The recruitment period is set at 12 months. The study was designed as a single-center study, as our tertiary hospital conducts the follow-up for the majority of patients with spina bifida in our country.

Our protocol has been developed using the *STROBE (Strengthening the Reporting of Observational Studies in Epidemiology)* guidelines, which provide a set of recommendations to ensure the transparent and comprehensive reporting of observational research.

### 2.3. Settings and Study Population

This single-center prospective cross-sectional study will include female patients with a documented diagnosis of spina bifida who are under follow-up at the Spina Bifida and Congenital Uropathy Clinic and Day Hospital at Policlinico A. Gemelli. Study personnel will recruit all eligible girls with a spina bifida diagnosis currently undergoing follow-up at the clinic and day hospital, pre-screened based on the inclusion and exclusion criteria outlined below.

Our study protocol has been reviewed and supervised by the Ethics Committee of the *Fondazione Policlinico Universitario Agostino Gemelli IRCCS* and Comitato Etico Territoriale Regione Lazio Area 3 (studio No. Profit ID CINECA 7629). Informed consent was obtained from all subjects involved in the study. Written informed consent has been obtained from the patients to publish this protocol paper. Adult patients and the parents of minor participants will be informed of the study’s purpose and will sign informed consent forms. Participants may choose to withdraw from the study at any time by notifying the Principal Investigator. We estimate to recruit a sample of at least 30 girls, prospectively enrolled during routine follow-up visits scheduled for patients with spina bifida.

### 2.4. Inclusion Criteria

- Age between 12 and 18 years;
- Onset of pubertal development with the appearance of menarche and/or full pubertal stage;
- Documented diagnosis of spina bifida;
- Signed informed consent to participate in the study.

### 2.5. Exclusion Criteria

- Use of oral combined estrogen–progestin contraceptives within three months prior to evaluation;
- Use of insulin-sensitizing medications within three months prior to evaluation;
- Use of folic acid within three months prior to evaluation;
- Known endocrine disorders;
- Known immunologic disorders;
- Known infectious diseases;
- Known oncological condition, current or previous;
- Lack of signed informed consent.

In the case of patients with a pre-existing diagnosis of PCOS at the time of screening, to prevent potential bias in the estimation of PCOS prevalence despite the presence of certain exclusion criteria (criteria 1 and 2), we included these patients in a parallel subgroup analysis. Furthermore, to improve the accuracy of our results, we plan to estimate the PCOS prevalence by imputing the data for these patients, assigning them a diagnostic weight based on the clinical phenotype [36] of those participants who successfully completed the study.

## 3. Study Variables and Timeline

All patients will undergo a multidisciplinary evaluation as outlined below:

### 3.1. First Day Hospital Visit

Upon the first visit to the Spina Bifida and Congenital Uropathy Day Hospital at Policlinico A. Gemelli, patients will undergo pre-enrollment and provide informed consent. During this day hospital visit, the following evaluations will be conducted:

- Medical History: A comprehensive medical history will be taken, covering both recent and past physiological and pathological aspects;
- Gynecological History: A general gynecological history will be collected;
- General Pediatric Assessment: Including a physical examination and blood pressure assessment;
- Endocrinological Assessment: This includes anthropometric measurements (BMI, waist-to-hip ratio, and abdominal circumference) and an evaluation of hypertrichosis and hirsutism risk factors using the modified Ferriman–Gallwey scale [37]. The auxological and laboratory parameters will be standardized according to Italian population percentiles or WHO percentiles for foreign patients [38];
- Blood Tests: Comprehensive blood exams will be conducted, including of complete blood count, thyroid-stimulating hormone (TSH), free triiodothyronine (FT3), free thyroxine (FT4), anti-thyroglobulin and anti-thyroid peroxidase antibodies, anti-Müllerian hormone (AMH), vitamin D, glucose, insulin, glycated hemoglobin (HbA1c), creatinine, glutamate oxaloacetate transaminase (GOT), glutamate pyruvate transaminase (GPT), total cholesterol, high-density lipoprotein (HDL), low-density lipoprotein (LDL), triglycerides, follicle-stimulating hormone (FSH), luteinizing hormone (LH), estradiol (E2), prolactin (PRL), dehydroepiandrosterone sulfate (DHEAS), androstenedione, testosterone, free testosterone index, sex hormone-binding globulin (SHBG), 17-hydroxyprogesterone, inositol, and homocysteine. Based on these results, indirect indices such as the homeostasis model assessment of insulin resistance (HOMA-IR) will be calculated;
- Pelvic Ultrasound (transvaginal/transabdominal): To assess ovarian morphology;
- Follow-up Blood Tests: To be conducted between days 3 and 10 of the spontaneous menstrual cycle, including of follicle-stimulating hormone (FSH), luteinizing hormone (LH), estradiol (E2), prolactin (PRL), dehydroepiandrosterone sulfate (DHEAS), androstenedione, testosterone, free testosterone index, sex hormone-binding globulin (SHBG), inositol, homocysteine, and 17-hydroxyprogesterone.

All the hematochemical and hormonal tests considered in our study will be analyzed according to the national standardized reference methodologies. Regarding the hormonal panel, testosterone and androgens, in general, will be analyzed using mass spectrometry (LC-MS/MS), which is currently the gold standard and the most accurate method for the analysis of steroid hormones. In the case of suspected insulin resistance (defined as a basal insulin level greater than 15, or an HOMA-IR greater than 2.5), patients will undergo a diagnostic OGTT to assess dynamic insulin secretion and, if necessary, initiate appropriate therapy.

A preliminary diagnosis of PCOS will be made based on the endocrinological evaluation and clinical presentation, to be confirmed via endocrine-metabolic tests and pelvic ultrasound, a standardized technique to assess ovarian morphology and the various associated pathological conditions [39,40]. PCOS diagnosis will be established following the exclusion of other endocrine disorders (e.g., adrenal hyperplasia, androgen-secreting tumors, Cushing’s syndrome, or acromegaly) and in accordance with the Rotterdam Consensus criteria [12,13], which require at least two of the following:

1. Oligo/anovulation;
2. Clinical (e.g., acne, hirsutism, or alopecia) and/or biochemical hyperandrogenism (elevated androgen levels);
3. Polycystic ovarian morphology on ultrasound (bilaterally normal or enlarged ovaries with at least 10 follicles of 2–8 mm in diameter, with or without an increased stroma-to-total ovarian area ratio).

Clinical hyperandrogenism was assessed through a standardized evaluation of hirsutism and acne, in accordance with the Rotterdam diagnostic criteria for PCOS. Hirsutism was quantified using the modified Ferriman–Gallwey (mFG) scoring system [37], which evaluates terminal hair growth in nine androgen-sensitive body areas; a score of ≥8 was considered indicative of clinical hirsutism. Acne severity was graded using a validated scale that takes into account the number, type (comedonal, papular, pustular, or nodular), and distribution of lesions. Both assessments were performed by trained clinicians to ensure consistency and reduce inter-observer variability. When present, androgenic alopecia was also noted as a potential marker of hyperandrogenism, although it was not used in isolation for diagnostic purposes. Oligo/anovulation was assessed according to menstrual cycle characteristics in relation to gynecological age, defined as the time since menarche. In line with current international recommendations, menstrual irregularity was considered physiological within the first year post menarche. Between one and three years after menarche, cycles longer than 45 days or fewer than eight cycles per year were classified as oligo/anovulatory. Beyond three years from menarche, persistent menstrual cycles exceeding 35 days or less than eight cycles per year were considered abnormal and consistent with oligo/anovulation. Detailed menstrual histories were obtained during clinical interviews to ensure accurate classification.

The Rotterdam criteria are routinely used in our center for the diagnosis of PCOS in young and adolescent girls, as they are incorporated into our institutional protocols. However, it is well recognized that these criteria were originally developed for adult women, and features such as irregular menstruation, cystic acne, mild hyperandrogenism, and multifollicular ovarian morphology may also occur as part of normal pubertal development [41]. Despite these limitations, the Rotterdam criteria remain the most widely accepted and reliable diagnostic tools for PCOS in adolescents to date [13]. In individuals under 18 years of age, diagnosis was approached with caution and aligned with international consensus guidelines, which recommend that PCOS in adolescence be diagnosed only when both persistent oligo/anovulation (assessed in relation to gynecological age) and clinical and/or biochemical hyperandrogenism are present, while excluding other causes of androgen excess. Importantly, polycystic ovarian morphology was not always considered a diagnostic criterion in this age group due to its low specificity in the early postmenarchal years. Therefore, the diagnosis of PCOS requires a multidisciplinary evaluation, even in the presence of positive Rotterdam criteria, incorporating the interpretation of pelvic ultrasound findings, baseline hormonal and biochemical profiles, and longitudinal follow-up. This comprehensive approach is essential, as several endocrine disorders of adolescence—such as non-classic congenital adrenal hyperplasia—or normal pubertal development can mimic the clinical presentation of PCOS [41].

### 3.2. Second Day Hospital Visit

For patients with a confirmed diagnosis of PCOS, a second day hospital visit will be scheduled. During this visit, further phenotypic characterization will be performed based on clinical, laboratory, and imaging findings. The classical phenotype will be associated with the presence of hyperandrogenism and oligo/anovulation, regardless of ultrasound findings.

During this visit, the following assessments will be conducted:

- General Pediatric Assessment.
- Endocrinological Assessment: Based on test results, confirmation of PCOS diagnosis and phenotype determination will be conducted. If deemed appropriate based on general bloodwork and glucose tolerance test results, treatment with insulin-sensitizing agents may be recommended.

Regarding the clinical utility of the data collected, the information extrapolated from our prospective study may be used to estimate the prevalence of PCOS in girls and young women with spina bifida. If the prevalence rate is found to be high, our data could be employed to modify the current recommendations for the multidisciplinary follow-up of these patients, potentially introducing early screening for PCOS in female patients.

## 4. Statistical Analysis

Given the lack of existing studies on the incidence of PCOS in patients with spina bifida, this study could be classified as a pilot study. Therefore, a formal sample size calculation is not necessary. However, based on the projected recruitment period and the number of patients observed in our tertiary center, which follows the majority of patients with spina bifida in our country, a sample of approximately 30 patients is expected to be enrolled.

Regarding the uniformity of the parameters, all variables will be analyzed considering the z-scores and percentiles standardized for the Caucasian population. In the interpretation of pelvic ultrasounds, we will use the parameters validated by the SIEDP, our national reference pediatric endocrinology society. Insulin resistance and glyco metabolic parameters will be interpreted according to the WHO guidelines.

If the recruited sample size allows, we will perform a subgroup analysis by distinguishing the different clinical phenotypes of PCOS, comparing pelvic ultrasound findings and laboratory results across the various groups.

Descriptive analysis with standard methods will be employed to measure the primary and secondary outcomes. Continuous variables in the two study groups will be compared using Student’s *t*-test or the Mann–Whitney U test, as appropriate, while categorical variables will be compared using the chi-squared test or Fisher’s exact test. To test for non-inferiority with a pre-specified margin of 10 percentage points, a generalized linear regression model with a logarithmic link and binomial distribution will be used to report risk differences. All hypotheses will be tested using two-tailed tests with a significance level of 0.05.

## 5. Discussion

Although the incidence of spinal dysraphisms has significantly decreased due to prevention strategies and the possibility of prenatal surgical correction [1,2], it is important not to overlook the children and young adults affected by this rare disease, which, if not properly managed, compromises the patients’ quality of life [42]. Ambulatory and urinary issues represent the major comorbidities of this particular condition [43]; one of the first studies on the prevalence of spinal dysraphisms [44] highlighted that in adult patients with lower urinary tract dysfunction, occult spina bifida and other spinal dysraphisms not evident upon objective evaluation may be a frequent cause of neurophysiological abnormalities. Given the possibility of the in utero surgical correction of spinal dysraphisms [44,45,46,47], it is possible that in the future, a protocol similar to ours could also include patients with prenatal correction, studying any differences between patients with pre- and postnatal surgical correction.

Our research protocol has some limitations. Despite the large number of patients followed in our center, the monocentric nature of the study may limit the generalizability of our results to the general population. Additionally, it is possible that patients classified by us as having PCOS may not actually have a coded diagnosis, as our criteria, when applied to girls and young adolescents, are only suitable in the presence of clear signs of hyperandrogenism [45,46,47]. Although lifestyle and dietary factors have been shown to influence the clinical presentation of polycystic ovary syndrome, particularly in adolescents, our study did not include an analysis of dietary parameters or lifestyle behaviors. Nayak et al. reported that women with PCOS, irrespective of body mass index, exhibited lower levels of physical activity and a higher consumption of processed foods and sugar-sweetened beverages, which could exacerbate the condition [47]. The lack of such data in our current analysis is a limitation, as these factors may contribute significantly to the metabolic and hormonal disturbances observed in PCOS. Future studies would benefit from incorporating a detailed assessment of lifestyle and dietary habits to better understand their role in the management and progression of PCOS in adolescents.

Despite these limitations, this is the first study addressing the potential correlation between PCOS and spina bifida, and therefore, our results will contribute valuable information regarding the multidisciplinary management of these patients.

## 6. Conclusions

Our study methodology is robust and recommended for research focusing on vulnerable populations, such as pediatric patients with spina bifida and young patients with PCOS. The study will collect comprehensive data that can be translated into evidence-based recommendations to address the complex health needs of children and young adults with a prior diagnosis of myelomeningocele and concurrent PCOS.

## Data Availability

The datasets generated and/or analyzed during the current study are available from the corresponding author upon reasonable request. Due to privacy and ethical considerations, access to personal data may be restricted. Aggregated data and results supporting the findings of this study are available in the publication and can be provided upon request to qualified researchers, following appropriate ethical review and approval.
